# ChiMera: an easy to use pipeline for bacterial genome based metabolic network reconstruction, evaluation and visualization

**DOI:** 10.1186/s12859-022-05056-4

**Published:** 2022-11-30

**Authors:** Gustavo Tamasco, Manish Kumar, Karsten Zengler, Rafael Silva-Rocha, Ricardo Roberto da Silva

**Affiliations:** 1 Ribeirão Preto School of Medicine (FMRP), University of São Paulo (USP), Ribeirão Preto, SP Brazil; 2 Department of Pediatrics, University of California, San Diego, 9500 Gilman Drive, La Jolla, CA 92093-0760 USA; 3 Department of Bioengineering, University of California, San Diego, La Jolla, CA 92093-0412 USA; 4 Center for Microbiome Innovation, University of California, San Diego, 9500 Gilman Drive, La Jolla, CA 92093-0403 USA; 5 School of Pharmaceutical Sciences of Ribeirão Preto, University of São Paulo, Ribeirão Preto, SP Brazil

**Keywords:** Genome-scale metabolic model, GSMR, Metabolic engineering, Metabolic network visualization, Flux balance analysis

## Abstract

**Background:**

Genome-scale metabolic reconstruction tools have been developed in the last decades. They have helped to reconstruct eukaryotic and prokaryotic metabolic models, which have contributed to fields, e.g., genetic engineering, drug discovery, prediction of phenotypes, and other model-driven discoveries. However, the use of these programs requires a high level of bioinformatic skills. Moreover, the functionalities required to build models are scattered throughout multiple tools, requiring knowledge and experience for utilizing several tools.

**Results:**

Here we present ChiMera, which combines tools used for model reconstruction, prediction, and visualization. ChiMera uses CarveMe in the reconstruction module, generating a gap-filled draft reconstruction able to produce growth predictions using flux balance analysis for gram-positive and gram-negative bacteria. ChiMera also contains two modules for metabolic network visualization. The first module generates maps for the most important pathways, e.g., glycolysis, nucleotides and amino acids biosynthesis, fatty acid oxidation and biosynthesis and core-metabolism. The second module produces a genome-wide metabolic map, which can be used to retrieve KEGG pathway information for each compound in the model. A module to investigate gene essentiality and knockout is also present.

**Conclusions:**

Overall, ChiMera uses automation algorithms to combine a variety of tools to automatically perform model creation, gap-filling, flux balance analysis (FBA), and metabolic network visualization. ChiMera models readily provide metabolic insights that can aid genetic engineering projects, prediction of phenotypes, and model-driven discoveries.

**Supplementary Information:**

The online version contains supplementary material available at 10.1186/s12859-022-05056-4.

## Background

Genome-scale metabolic reconstructions (GSMRs) are essential tools in system biology [1]. Over the last 30 years, GSMRs provided researchers with the necessary tools to gain insight into microbial evolution, network interaction, genetic engineering, drug discovery, prediction of phenotypes, and model-driven discoveries [2]. However, the generation of a precise genome-scale metabolic model can be very complex and time-consuming, requiring several steps [3]. The process starts with genome annotation and assembly of all associated known metabolites and reactions, which creates an initial metabolic reconstruction to build a draft model. Several rounds of manual curation and evaluation of the present genes, reactions, and compounds are necessary to create a high-quality metabolic model. After these steps, one needs to set a biological objective function in the model (e.g., biomass function) followed by the conversion to a mathematical formulation known as the stoichiometric matrix (S-matrix), which is a computer-readable core part of the model. The S-matrix is used to simulate the models performing flux balance analysis (FBA) and growth predictions [3]. Other steps, such as gap-filling and stoichiometric balance, may also be necessary, increasing the complexity of the process.

Recently, several tools such as AureMe [4], Pathway Tools [5], RAVEN [6], Model SEED [7] and Merlin [8] were developed to assist with model creation [9]. A few of those tools were designed to handle specific processes. CarveMe [10] is a command-line tool that deals with the initial phase of model creation and gap-filling. Cobrapy [11] can convert draft models into an S-matrix and perform FBA analysis using optimized algorithms. Escher [12] offers a fully customizable suite for pathway visualization. However, the latter tools require familiarity with command-line interfaces and programming [13]. They also have their peculiarities, demanding time and knowledge from users to perform the analysis. Therefore, the use of those tools by non-bioinformaticians can be challenging, and the number of steps required to build initial models precludes their usage in large-scale projects, which may include the development of models for hundreds of genomes.

**Fig. 1:**
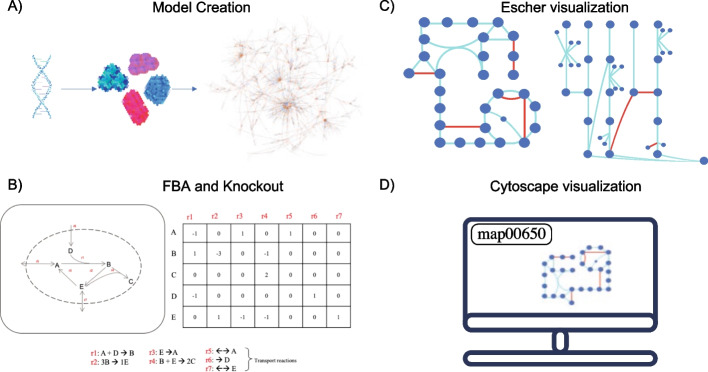
Schematic of processes automated by ChiMera. We use automation to compile several tasks and create an organism specific model based on the genomic data. **A** Model creation using CarveMe pruning algorithm requires the user to provide only a protein annotation file based on the genome. **B** Model conversion to a stoichiometric matrix to perform flux balance analysis and gene and reaction knockouts. **C** Representation of pre-defined Escher maps, reactions depicted in blue were detected in the model, red ones were absent. **D** Users can use the Cytoscape search bar to select specific pathways

Here we present a novel tool named ChiMera, which compiles widely used tools for genome-scale metabolic modeling in a single pipeline. ChiMera uses a protein sequence as input (*.faa file) and creates a model based on a highly curated universal model [10]. The resulting draft model can be used for FBA and growth predictions, knockout simulations, and pathway visualizations (Fig. 1). To evaluate models generated by ChiMera, using CarveMe algorithm, we compared several aspects of model completion with manually curated models from the literature. We also compared the predicted growth values with experimental data to ensure model accuracy.

## Implementation

### General ChiMera structure

**Fig. 2:**
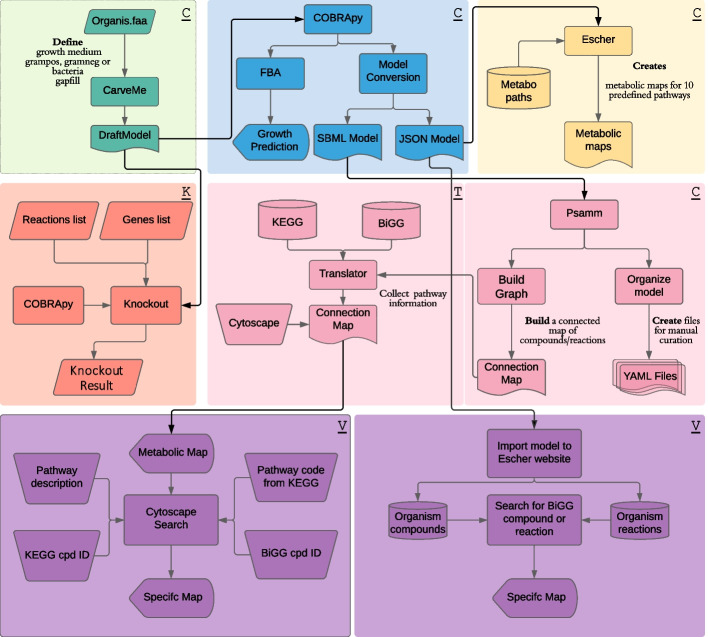
Flow chart of ChiMera processes. ChiMera has 3 submodules that can be used separately. The ones signed with “C” are part of the core module, which performs model creation, evaluation and creation of visualization files. “T” represents the translator module that adds KEEG pathway information to the compounds in the edges file. The file can be loaded into Cytoscape or Gephi for visualization. “K” represents the knockout module, which performs gene and reaction knockouts. “V” represents the use of outputs created by ChiMera to create custom maps by third-party tools based on user needs

ChiMera uses automation algorithms to combine three main steps in GSMR, i.e. model creation and gap-filling, FBA, and pathway visualization. The tool also includes a submodule that enables users to perform in silico gene and reaction essentiality screening based on FBA. All these functions are modular and compatible with further expansions of ChiMera. (Fig. 2).

### Model creation and gap-filling

We use CarveMe (v1.5.1) in the reconstruction module of ChiMera. The initial draft model is created based on the protein sequence file (*.faa file) provided by the user. During the reconstruction process, ChiMera uses the CarveMe gap-filling algorithm to add missing reactions based on a given growth media. This process uses genomic evidence to ensure that the model will be able to predict growth in the condition. CarveMe uses a top-down approach in a pre-built reference and manually curated universal model [10]. It applies a pruning algorithm that removes reactions not supported by genomic evidence, generating an organism-specific model based on highly curated data [10].

CarveMe utilizes five predefined media: LB, anaerobic LB, M9, anaerobic M9, and M9 using glycerol as a carbon source [10]. A tab-separated file, containing a new media composition can also be used to reconstruct the model.

### S-matrix construction and initial FBA

We used COBRApy (v0.22.1) to convert the initial draft into an S-matrix and perform FBA analysis [11]. Growth, uptake, and secretion metrics are displayed in the command line for the user and stored in a file. The tool is also used in the knockout module, enabling users to perform targeted single or double gene/reaction knockout. A file with the gene name or reaction name needs to be provided by the user. Additionally, we included an option to perform a single gene/reaction knockout in the whole model. This provides the resulting growth upon knockout for each gene/reaction in the organism.

### Visualization of the metabolic maps

ChiMera converts the initial SBML model to 2 different model formats: JSON, and YAML. These model formats are compatible with the majority of currently available tools.

We performed transformations in the JSON model to enable compatibility between Escher maps and the user model. We developed *in-house* algorithms to automate the generation of metabolic maps based on Escher (v1.7.3) [12]. Ten predefined pathway maps are pre-loaded in this module. Users can also provide custom JSON maps of desired pathways to check if they are present in the target organism. A video demonstration is provided for users’ benefit to understand how to add new Escher maps to ChiMera [14, 15]. The pipeline uses the model data to evaluate reactions and compounds present in the organism, creating customizable HTML maps that can be edited by the user.

Furthermore, we developed a second visualization module that creates files compatible with graphical visualization tools. ChiMera automated the use of PSAMM (v1.1.2), converting the model to a graphical representation [16]. The graphical representation only contains information about the connection between nodes (compounds) and edges (reactions). Users can use the "harvest path" submodule to convert BiGG ids to KEGG ids. This submodule collects information on the pathways that the compounds participate in. This approach creates a graphical representation file with pathway information that can be loaded into Cytoscape [17]. The pathway information can be used to select specific maps from the whole network [18].

### Genome selection and model generation

For demonstrating functionality of ChiMera, we selected two well-studied Gram-negative bacteria (*Pseudomonas putida* KT2440 and *Escherichia coli*) and one Gram-positive bacterium (*Bacillus subtilis*). Protein sequence files were downloaded from NCBI under the accession numbers NC_002947.4, NZ_CP020543.1, and AL009126.3. These genomes were used to generate the models, visualizations and knockouts. Further, these ChiMera models were compared with manually curated models from the BiGG database, iJN1463 (*P. putida*), iEC1344_C (*E. coli*), and iYO844 (*B. subtilis*).

### Model evaluation

We performed basic tests to check the correctness of the models using MEMOTE, which benchmarks the model by applying consensus tests based on model annotation, biomass composition, network topology, stoichiometry, and biomass composition and consistency [19]. We also performed gene essentiality benchmarking to assay the effect of a single-gene deletion. The media composition was defined as M9 minimal medium for all organisms. To calculate the performance metrics we measured the reconstruction module ability to correctly assign a gene as non-essential or essential. Predicted outcomes were compared to the curated models (Additional file 2: Table S1). Published experimental mutant knockout data was used to evaluate the predictions [20–22] (Additional file 2: Table S2). To examine the prediction capabilities of produced models and curated ones, we simulated their behavior using different carbon sources that were previously experimentally tested in the laboratory for growing *B. subtilis*, *E. coli*, and *P. putida* [23–28]. Except for the carbon source, the uptake rates of other nutrients were kept constant in each simulation. Each carbon source was constrained using lower and upper bounds of -10 and 0. A list of carbon sources is provided in Additional file 2: Tables S3, S4, and S5.

We also compared the sets of compounds for each organism in automated and manual reconstructions. The unique compounds of each organism-specific model were selected, and their metabolic role was inferred using the ChiMera path harvest submodule. The metabolic profile from curated and automatically generated models was compared using Principal Component Analysis and Hierarchical Clustering of the 30 most frequently detected pathways.

### Performance metrics

We used 6 different performance metrics to compare the gene essentiality predictions, the experimental data was used as ground thruth.

$$\begin{gathered} {\text{Precision}}:{\text{ TP}}/\left( {{\text{TP}} + {\text{FP}}} \right) \hfill \\ {\text{Sensitivity}}:{\text{ TP}}/\left( {{\text{TP}} + {\text{FN}}} \right) \hfill \\ {\text{Specificity}}:{\text{ TN}}/\left( {{\text{TN}} + {\text{FP}}} \right) \hfill \\ {\text{Accuracy}}: \, \left( {{\text{TP}} + {\text{TN}}} \right)/\left( {{\text{TP}} + {\text{FP}} + {\text{FN}} + {\text{TN}}} \right) \hfill \\ {\text{Negative Predictive Value}}\left( {{\text{NVP}}} \right):{\text{ TN}}/\left( {{\text{TN}} + {\text{FN}}} \right) \hfill \\ {\text{F score}}:{2 }* \, \left( {\left( {{\text{Precision }}*{\text{Sensitivity}}} \right)/\left( {{\text{Precision}} + {\text{Sensitivity}}} \right)} \right) \hfill \\ \end{gathered}$$

where TP = True Positive, TN = True Negative, FP = False positive and FN = False Negative predictions.

### ChiMera environment and user interface

ChiMera is a portable command-line-based tool. The source code, along with complete documentation of its utilization and examples of inputs are available at https://github.com/tamascogustavo/chimera, https://sourceforge.net/projects/chimera-gsmr/ [29].

## Results

### Key capabilities of ChiMera

**Fig. 3:**
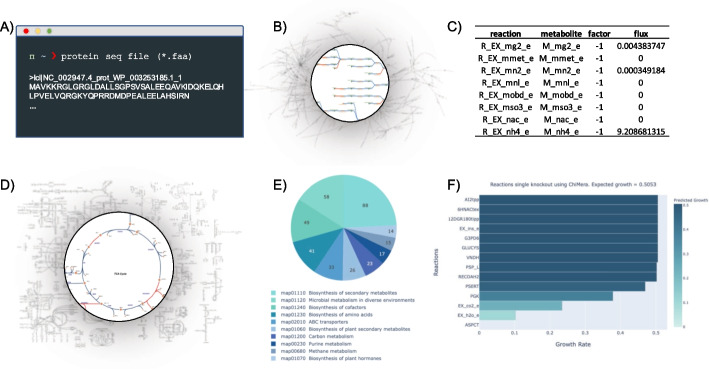
ChiMera results example for the model reconstruction of *P. putida.* **A** Schematic representation of initial protein sequence file required for ChiMera to perform reconstruction, model evaluation, and visualization. **B** SBML model representation. **C** Partial output of Flux Balance Analysis simulation for the GSMR. **D** Metabolic maps generated by Escher during ChiMera visualization module execution. Here we highlighted the TCA cycle. Blue reactions were detected in the model, the red ones indicate absence. **E** ChiMera enriched pathways result. Only the ten most frequently detected pathways are displayed. **F** Barplot of ChiMera single reactions knockout module. ChiMera was used to evaluate the impact of reaction knockout. The units are in g[CDW]/h

ChiMera was implemented in python v3.7 and its dependencies are freely available. There are four main functions of ChiMera: model creation, flux balance analysis and growth prediction, metabolism visualization, and knockout evaluation. ChiMera relies on CarveMe to create an organism-specific model. A curated model is pruned to produce a draft model containing thermodynamic balanced reactions and elemental balanced metabolites using a protein sequence file as input (Fig. 3A). The draft model has three compartments, i.e. the cytosol, periplasm, and extracellular space. During the reconstruction, the user can select one of the five predefined media, or can build a specific media composition. Subsequently gap-filling based on the genomic evidence is performed to ensure that the organism-specific model can grow under the provided or experimentally-tested growth conditions. If the model is not able to grow in the given medium, a message is displayed, informing that the gap-filing has failed to enable growth. We recommend M9 minimal media as the base of new formulations, avoiding missing precursors that lead to gap-filling errors (Fig. 3B).

Next, the organism-specific model is automatically converted to a S-matrix, using COBRApy. The biomass-producing reaction, which contains the precursors like carbohydrate, protein, lipids, and energy molecules balanced for producing one gram of biomass, is set as the biological objective function for performing FBA (Fig. 3C). The fluxes of uptake and secretion based on the media, along with growth value are displayed to the user (Additional file 1: Fig. S1). Subsequently, the model is converted to a JSON format, used to produce predefined metabolic maps based on Escher (Fig. 3D) (Additional file 1: Fig. S2). However, users can design specific maps and add them to ChiMera pipeline (Additional file 1: Fig. S3). The model is also converted to YAML format, which is used by the PSAMM *findprimalpairs* algorithm to break down the GSMR into connections between metabolites (nodes) and reactions (edges).

The output of PSAMM can be directly loaded into Cytoscape [30], producing a visualization of the entire reconstruction. Users can also use the ChiMera translator submodule, to add pathway information to the file, enabling a targeted search of pathways in Cytoscape (Additional file 1: Fig. S4).

ChiMera also produces a broader view of the target metabolism (Fig. 3E). The compounds detected in the model have their metabolic association collected from the KEGG database, and the information of the most frequently detected pathways is used to create an interactive plot (Additional file 1: Fig. S5).

To allow ChiMera's flexibility and modularity, users can also provide a pre-built model with the protein sequence file, which should hold the same prefix, directly performing FBA analysis and construction of the pathway maps. Documentation is provided to ensure that the annotations of the model or the presence of extra compartments are compatible with PSAMM, to generate the Cytoscape compatible file. We provide tutorials on how to use ChiMera output files to build custom maps for any organism (see Materials and Methods).

The knockout module of ChiMera is dependent on COBRApy [11]. Here, we implemented a function that enables the user to provide a file containing a list of genes or reactions to be silenced. This module can perform single or double targeted deletions (Fig. 3F). Results are displayed in the command line (Additional file 1: Fig. S6). The user can also perform gene essentiality analysis for the whole model, identifying the impact of silencing the genes/reactions on the growth under given growth conditions (Additional file 2: Table S6).

### Comparison with manually curated models

**Fig. 4:**
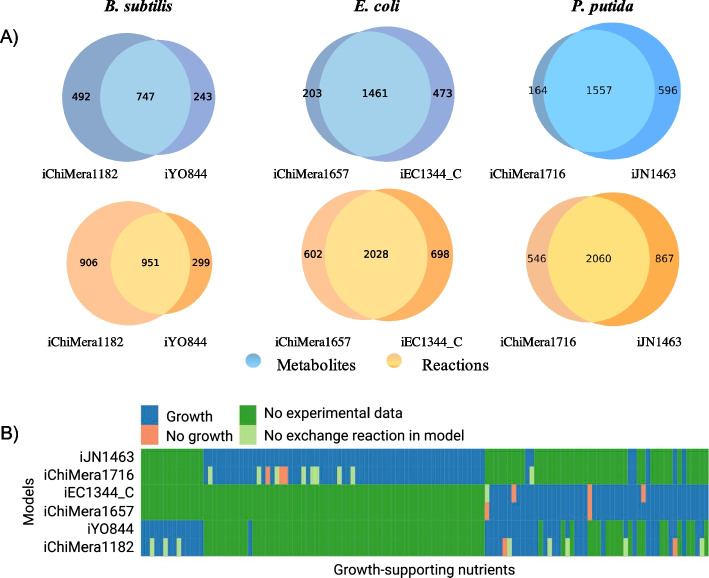
Comparison between ChiMera and manually curated models of *B. subtilis*, *E. coli*, and *P. putida*. **A** Venn diagram of reactions and metabolites sets. Reactions and compound sets from ChiMera (iChiMera1182, iChiMera1657, and iChiMera1716) and manually curated models (iYO844, iEC1344_C, and iJN1463) were compared to identify the intersection of the model features. Model-specific information is also depicted. **B** Heatmap illustrates predicted growth using ChiMera and manually curated models on different experimentally tested growth environments. The models of *B. subtilis*, *E. coli*, and *P. putida* were used to simulate growth on 46, 50, and 70 carbon sources, respectively. These carbon sources and in silico growth rates can be seen in Additional files 2: Tables S4, S5, and S6. The data in heatmap was clustered based on rows and columns

We compared sets of metabolites and reactions included in the models with those present in manually curated models. ChiMera1716 (*P. putida*) and iJN1463 models shared 68% of their metabolites and 60% of their reactions. Similar values were observed for iChiMera1657 (*E. coli*) and iEC1344_C models. iChiMera1182 (*B. subtilis*) shared 50% of its metabolites and 44% of its reactions with iYO844 (Fig. 4 A).

We further investigated the presence of exclusive compounds in manually curated models and automatically generated models. The first component of the PCA analysis separated the dataset into gram-positive and gram-negative reconstructions, in the second component, the models were divided based on the reconstruction method. Hierarchical Clustering of the 30 most frequently detected pathways produced similar results, except for the gram-positive reconstructions, which swapped. These results suggest that the reconstruction method has more impact on the model draft. (Additional file 1: Fig. S7).

We also performed a more comprehensive comparison of model features based on MEMOTE metrics [19]. The overall score of ChiMera models is comparable to the manually curated models. Moreover, models produced by CarveMe algorithm have a lower number of blocked reactions, orphan and dead-end metabolites. Curated models had a higher presence of missing essential precursors in the biomass function, which can lead to unrealistic growth predictions (Table 1).

**Table 1:** MEMOTE evaluation metrics

Model ID	Balance metrics			Biomass constitution			Network topology	
	Stoichiometric	Mass	Charge	Biomass constitution	Missing precursors in biomass	Blocked reactions	Orphan metabolites	Dead-end metabolites
*P. putida* iChiMera1716	99.8	99.9	80.2	1.00	1	19	0	0
iJN1463	0	99.6	99.7	0.98	2	247	56	85
*E. coli* iChiMera1657	99.6	99.9	83.2	1.00	1	18	0	1
iEC1344_C	100	100	74	1.54	30	0	0	1
*B. subtilis* iChiMera1182	100.0	99.9	82.1	1.03	1	54	1	1
iYO844	100.0	94.4	98.9	1.04	6	50	122	21

Parameters that can influence the precision of the predictions were selected to assay Chimera and BiGG curated models. Values in the range of 1±$1{0}^{-3}$ in Biomass Constitution are necessary to indicate a realistic biomass function

Next, we examined the prediction capabilities of models by comparing predicted growth with experimentally measured growth rates. Curated and non-curated models shared a close resemblance. Both sets of models were simulated using 46, 50, and 70 different carbon sources for *B. subtilis* [27, 28], *E. coli* [23, 24], and *P. putida* models [25], respectively (Additional file 2: Tables S4, S5 and S6). This analysis suggested that ChiMera models were able to perform comparably to manually-curated models. In comparison with manually-curated models, they predicted 96 to 100% accurate growth on different carbon sources (Fig. 4B).

### Gene essentiality metric evaluation

**Fig. 5:**
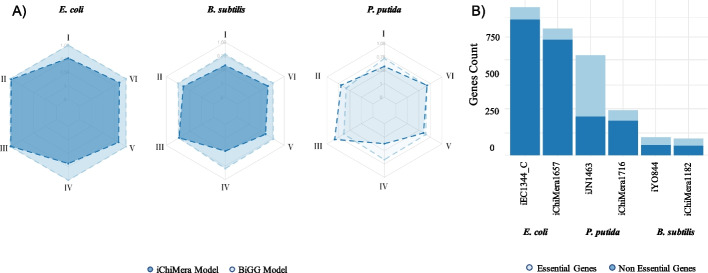
Gene essentiality metrics. Six metrics were selected to compare prediction capability from ChiMera and manually curated models. **A** Radarplot of Gene Essentiality metrics. **B** Stacked bar plot of gene essentiality classification according to presence in the genome

Before we evaluate the predictions of each model, gene datasets for each organism were normalized based on the weighted average of hits in the model (Fig. 5B). Model performance statistics were calculated by the ability to detect essential genes and non-essential genes, respectively (Additional file 2: Table S1).

ChiMera knockout module was used to perform the evaluation. The gene essentiality predictive metrics were higher in manually curated models. Comparing *P. putida* models, iChiMera1716 and iJN1463, we observed that the curated model had worse specificity and precision and better performance at the sensitivity and negative predictive value. For *E. coli*, the iEC1344_C had a perfect prediction on the dataset. The iChiMera1657 model was outperformed in sensitivity, negative predictive value, accuracy, and F1 score. For *B. subtilis*, we observed a better performance at specificity, negative predictive value, accuracy, and F1 score for iYO844 (Fig. 5A). ChiMera's models were outperformed in sensitivity and negative predictive value in all the comparisons. Metadata indicates that our models had a higher mislabeling of essential genes (Additional file 2: Table S1).

## Discussion

We introduce ChiMera, an automated, well-documented and easy-to-use command-line tool that enables researchers with limited knowledge of bioinformatics and computational biology to produce GSMRs. These reconstructions can be great tools to explore the metabolic potential of the target organisms. Gene essentiality modules within ChiMera can help researchers to understand the behavior of the organisms under diverse experimental conditions. The visualization modules facilitate the exploration of essential pathways, as well as the identification of unique pathways for non-model organisms. Collectively, the outcome provided by ChiMera assists researchers in understanding non-model organism metabolism and developing metabolic engineering approaches for model organisms.

ChiMera has similar goals to AuReMe and Merlin. These tools offer a custom workspace for the user, hence facilitating the construction of genome-scale models. AuReMe has its own data structure based on PADMet, and focuses on traceability of the reconstruction process, performing at its best if highly curated models are available [4]. There are several steps that the user can process, but it lacks visualization and knockout modules (Additional file 2: Table S7). AureMe performance was comparable to CarveMe in model creation [9]. Merlin offers a vast workspace for its users. Its graphical interface allows users to re-annotate genomes using BLAST or HMMER, and also integrate data from NCBI and KEGG to its draft model [8]. This tool is preferable for those focusing on manual curation of single organisms with expertise in metabolic engineering and model creation [9].

ChiMera inherits some pros and cons from CarveMe. The top-down approach based on a universally curated model generates draft reconstructions that share coverage of reactions and metabolites above 60% compared to highly curated models, suggesting a great alternative for the first model draft, before manual curation (Fig. 4A). ChiMera models are also valuable assets for those working with hundreds of genomes due to the easiness and speed of a draft construction, enabling researchers to evaluate multiple candidate models and choosing the best option for a manual curation if needed. We demonstrated that the ChiMera models can predict phenotypes comparable to manually curated models (Fig. 4B). We also observed good agreement in gene essentiality detection between ChiMera and manually curated models. Manually curated models mostly had higher prediction capabilities compared to ChiMera models (Fig. 5A). However, the differences were more accentuated for sensibility and negative predictive value where the metrics between ChiMera models and curated ones agreed 76% and 61%, respectively. For accuracy, ChiMera achieved 84% of the curated model predictions. Specificity and precision metrics were similar, with marginal advantage to ChiMera predictions. These inferences are held with a F-score of 91%. These results demonstrate that the choice to use CarveMe in the reconstruction module was advantageous in several aspects, ranging from draft models with resemblance to manually curated models, gap-filling based on higher genetic evidence, to fast performance [9].

ChiMera complements the reconstruction module based on CarveMe by adding a new visualization module that allows the user to have a comprehensive overview of the organism's metabolism. One can rely on the predefined maps or design specific maps using ChiMera outputs to suit their research needs [14, 15, 18]. We also provide models in different formats that enable compatibility with most of the tools used to create GSMR.

Finally, the implementation of FBA and knockout modules can help to elucidate ecological niches and the planning of knockout strategies (Fig. 3F). These modules can also assist in pathway engineering, identifying the best silencing strategies to deflect the metabolic flux to the desired metabolite. ChiMera archives all these functionalities in a modular and easy-to-use pipeline.

## Conclusion

ChiMera is a novel command-line tool that automatizes the usage of state-of-art GSMR tools, enabling biologists with little experience in model reconstruction to create ready-to-simulate genome-scale models. ChiMera contains submodules that enable users to investigate the metabolic pathways present in the target organism. Furthermore, the tool performs gene or reaction knockout simulations, facilitating the development of engineering strategies. To demonstrate the benefits of ChiMera, we compared gene essentiality and growth prediction capability of ChiMera models against well-curated models. As a result, ChiMera provides automatization of a unique set of tools, for biologists who are interested in genome-scale models, as well as for those interested in a more comprehensive understanding of an organism's metabolism.

### Availability and requirements

Project name: ChiMera.

Project homepage: https://github.com/tamascogustavo/chimera, https://sourceforge.net/projects/chimera-gsmr/

https://pypi.org/project/ChiMera-ModelBuilder/

Operating System(s): Mac Os, Linux.

Programming language: Python.

Other requirements: The tool has dependence on other thirst party software. All dependencies are handled during the installation and creation on an virtual environment.

License: GNU GLP 3.

Any restrictions to use by non-academic: ChiMera has no restriction, however CarveMe rely on IBM ILOG CPLEX Optimization Studio, that demands an academic license.

## Supplementary Information

**Additional file 1**. **Figs. S1–S7** ChiMera output examples and supplementary analysis.

**Additional file 2**. **Tables S1–S7** ChiMera supplementary tables and metadata.

## Data Availability

All data used to validate ChiMera is freely available and can be found in our GitHub: https://github.com/tamascogustavo/chimera. Tables and images in the Manuscript and in Supplementary are included within the paper data, and also available on our GitHub. The code is available on Github, PyPI, SourceForge and Zenodo. The version (ChiMera v2.0.1) used in this paper can be found on Zenodo under https://doi.org/10.5281/zenodo.6945772, GitHub release 2.0.1 or PyPI 2.0.15. Protein sequence files used to generate ChiMera models were downloaded from NCBI under the accession numbers NC_002947.4, NZ_CP020543.1, and AL009126.3. The manually curated models were downloaded from BiGG database under the following BiGG identifiers, iJN1463 (*P. putida* genome accession NC_002947.4), iEC1344_C (*E. coli* genome accession NZ_CP020543.1), and iYO844 (*B. subtilis* genome accession AL009126.3).

## Abbreviations

FBA: Flux balance analysis
GSMR: Genome scale metabolic reconstruction
S-matrix: Stoichiometric matrix

## References

[1] Monk J, Nogales J, Palsson BO (2014). Optimizing genome-scale network reconstructions. Nat Biotechnol.

[2] Bordbar A, Monk JM, King ZA, Palsson BO (2014). Constraint-based models predict metabolic and associated cellular functions. Nat Rev Genet.

[3] Thiele I, Palsson BO (2010). A protocol for generating a high-quality genome-scale metabolic reconstruction. Nat Protoc.

[4] Aite M, Chevallier M, Frioux C, Trottier C, Got J, Cortés MP (2018). Traceability, reproducibility and wiki-exploration for “à-la-carte” reconstructions of genome-scale metabolic models. PLoS Comput Biol.

[5] Karp PD, Paley SM, Midford PE, Krummenacker M, Billington R, Kothari A, et al. Pathway Tools version 24.0: Integrated software for pathway/genome informatics and systems biology. 2020. ArXiv: http://arxiv.org/abs/1510.03964.

[6] Agren R, Liu L, Shoaie S, Vongsangnak W, Nookaew I, Nielsen J (2013). The RAVEN toolbox and its use for generating a genome-scale metabolic model for *Penicillium chrysogenum*. PLoS Comput Biol.

[7] High-throughput generation, optimization and analysis of genome-scale metabolic models | Nat Biotechnol [Internet]. [cited 2022 Feb 7]. Available from: https://www.nature.com/articles/nbt.1672 10.1038/nbt.1672 20802497

[8] Capela J, Lagoa D, Rodrigues R, Cunha E, Cruz F, Barbosa A (2021). *merlin* v4.0: An updated platform for the reconstruction of high-quality genome-scale metabolic models. Bioinformatics.

[9] Mendoza SN, Olivier BG, Molenaar D, Teusink B (2019). A systematic assessment of current genome-scale metabolic reconstruction tools. Genome Biol.

[10] Machado D, Andrejev S, Tramontano M, Patil KR (2018). Fast automated reconstruction of genome-scale metabolic models for microbial species and communities. Nucleic Acids Res.

[11] Ebrahim A, Lerman JA, Palsson BO, Hyduke DR (2013). COBRApy: constraints-based reconstruction and analysis for python. BMC Syst Biol.

[12] King ZA, Drager A, Ebrahim A, Sonnenschein N, Lewis NE, Palsson BO (2015). Escher: a web application for building, sharing, and embedding data-rich visualizations of biological pathways. PLoS Comput Biol.

[13] Passi A, Tibocha-Bonilla JD, Kumar M, Tec-Campos D, Zengler K, Zuniga C (2022). Genome-scale metabolic modeling enables in-depth understanding of big data. Metabolites.

[14] Tamasco G. How to add new Escher maps to ChiMera [Internet]. [cited 2022 Jan 7]. Available from: https://www.youtube.com/watch?v=YeAczYRWLTI

[15] Tamasco G. Build your own metabolic map with Chimera outputs [Internet]. [cited 2022 Jan 7]. Available from: https://www.youtube.com/watch?v=XQRbSkvMpN4

[16] Steffensen JL, Dufault-Thompson K, Zhang Y (2016). PSAMM: a portable system for the analysis of metabolic models. PLOS Comput Biol..

[17] Cokelaer T, Pultz D, Harder LM, Serra-Musach J, Saez-Rodriguez J (2013). BioServices: a common Python package to access biological Web Services programmatically. Bioinformatics.

[18] Tamasco G. How to visualize ChiMera metabolic maps using Cytoscape [Internet]. [cited 2022 Jan 7]. Available from: https://www.youtube.com/watch?v=M7SNCnPwqF0

[19] Lieven C, Beber ME, Olivier BG, Bergmann FT, Ataman M, Babaei P (2020). MEMOTE for standardized genome-scale metabolic model testing. Nat Biotechnol.

[20] Turner KH, Wessel AK, Palmer GC, Murray JL, Whiteley M (2015). Essential genome of *Pseudomonas aeruginosa* in cystic fibrosis sputum. Proc Natl Acad Sci.

[21] Orth JD, Conrad TM, Na J, Lerman JA, Nam H, Feist AM, Palsson BØ (2011). A comprehensive genome-scale reconstruction of *Escherichia coli* metabolism—2011. Mol Syst Biol.

[22] Kobayashi K, Ehrlich SD, Albertini A, Amati G, Andersen KK, Arnaud M (2003). Essential *Bacillus subtilis* genes. Proc Natl Acad Sci.

[23] Monk JM, Koza A, Campodonico MA, Machado D, Seoane JM, Palsson BO (2016). Multi-omics quantification of species variation of *Escherichia coli* links molecular features with strain phenotypes. Cell Syst.

[24] Monk JM, Lloyd CJ, Brunk E, Mih N, Sastry A, King Z (2017). iML1515, a knowledgebase that computes *Escherichia coli* traits. Nat Biotechnol.

[25] Nogales J, Mueller J, Gudmundsson S, Canalejo FJ, Duque E, Monk J (2020). High-quality genome-scale metabolic modelling of *Pseudomonas putida* highlights its broad metabolic capabilities. Environ Microbiol.

[26] Henry CS, DeJongh M, Best AA, Frybarger PM, Linsay B, Stevens RL (2010). High-throughput generation, optimization and analysis of genome-scale metabolic models. Nat Biotechnol.

[27] Oh Y-K, Palsson BO, Park SM, Schilling CH, Mahadevan R (2007). Genome-scale reconstruction of metabolic network in *Bacillus subtilis* based on high-throughput phenotyping and gene essentiality data *. J Biol Chem.

[28] Henry CS, Zinner JF, Cohoon MP, Stevens RL (2009). iBsu1103: a new genome-scale metabolic model of *Bacillus subtilis* based on SEED annotations. Genome Biol.

[29] Tamasco G. ChiMera: an easy to use pipeline for genome based metabolic network reconstruction, evaluation and visualization [Internet]. Available from: 10.5281/zenodo.5720515 10.1186/s12859-022-05056-4 PMC9710178 36451100

[30] Shannon P, Markiel A, Ozier O, Baliga NS, Wang JT, Ramage D (2003). Cytoscape: a software environment for integrated models of biomolecular interaction networks. Genome Res.

