# Supplementary material for: ChiMera: an easy to use pipeline for bacterial genome based metabolic network reconstruction, evaluation and visualization
Source: BMC Bioinformatics. 2022 Nov 30;23:512. doi: 10.1186/s12859-022-05056-4 (PMC9710178; doi:10.1186/s12859-022-05056-4)
Supplement: Supplementary file 1 — Additional file 1. Figs. S1–S7 ChiMera output examples and supplementary analysis. [file 12859_2022_5056_MOESM1_ESM.docx]

**ChiMera: An easy to use pipeline for bacterial Genome Based Metabolic Network Reconstruction, Evaluation and Visualization**

Gustavo Tamasco^1^, Manish Kumar^2^, Karsten Zengler^2,3,4^, Rafael Silva Rocha^1^, Ricardo Roberto da Silva^25^

^1^Ribeirão Preto School of Medicine (FMRP) - University of São Paulo (USP) - Ribeirão Preto, SP, Brazil.

^2^Department of Pediatrics, University of California, San Diego, 9500 Gilman Drive, La Jolla, CA 92093-0760, USA

^3^Department of Bioengineering, University of California, San Diego, La Jolla, CA 92093-0412, USA

^4^Center for Microbiome Innovation, University of California, San Diego, 9500 Gilman Drive, La Jolla, CA 92093-0403, USA

^52^ School of Pharmaceutical Sciences of Ribeirão Preto, University of São Paulo, Ribeirão Preto, SP, Brazil

*** Correspondence:**

Gustavo Tamasco

gustavo.tamasco@usp.br

Supplementary Material

**Supplementary Figure 1:** ChiMera core module growth prediction output. The core module shows in the user screen the growth rate given the conditions provided to Chimera.

**Supplementary Figure 2:** Pre-defined map of carbon central metabolism in *E. coli*. Blue reactions were identified in the model. The reaction in red had no evidence detected in the model, or there was a mismatch on reactions ids. The map is a modified version of Escher default maps.


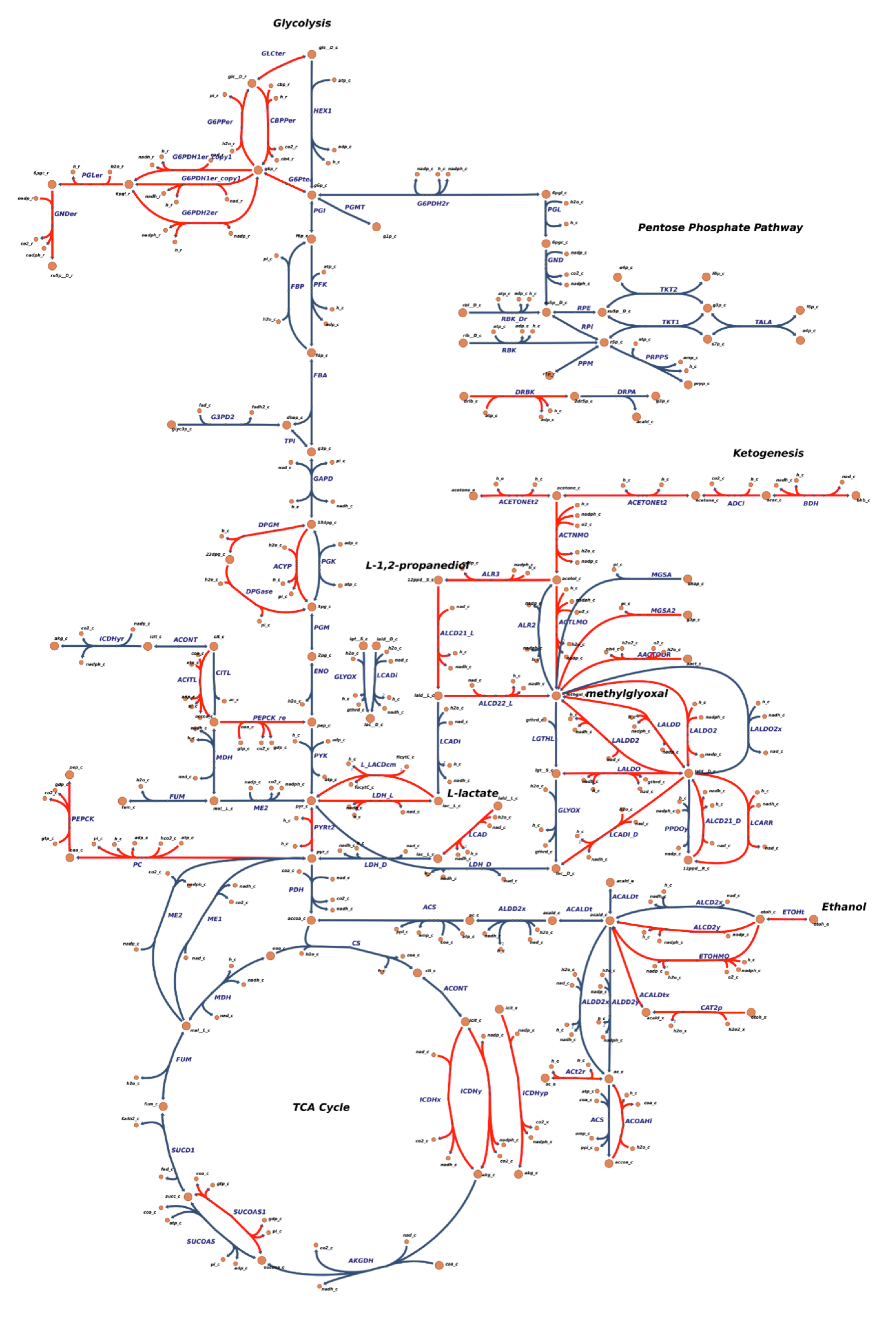


**Supplementary Figure 3:** Custom designed Escher maps for pentose phosphate and histidine biosynthesis. (A) These maps were designed based on the *E. coli* model inside Escher website. (B) The maps were included in the core module of ChiMera and used along the other ten maps. Blue and red indicate presence and absence of reactions in the model, respectively. The map is a modified version of Escher default maps.


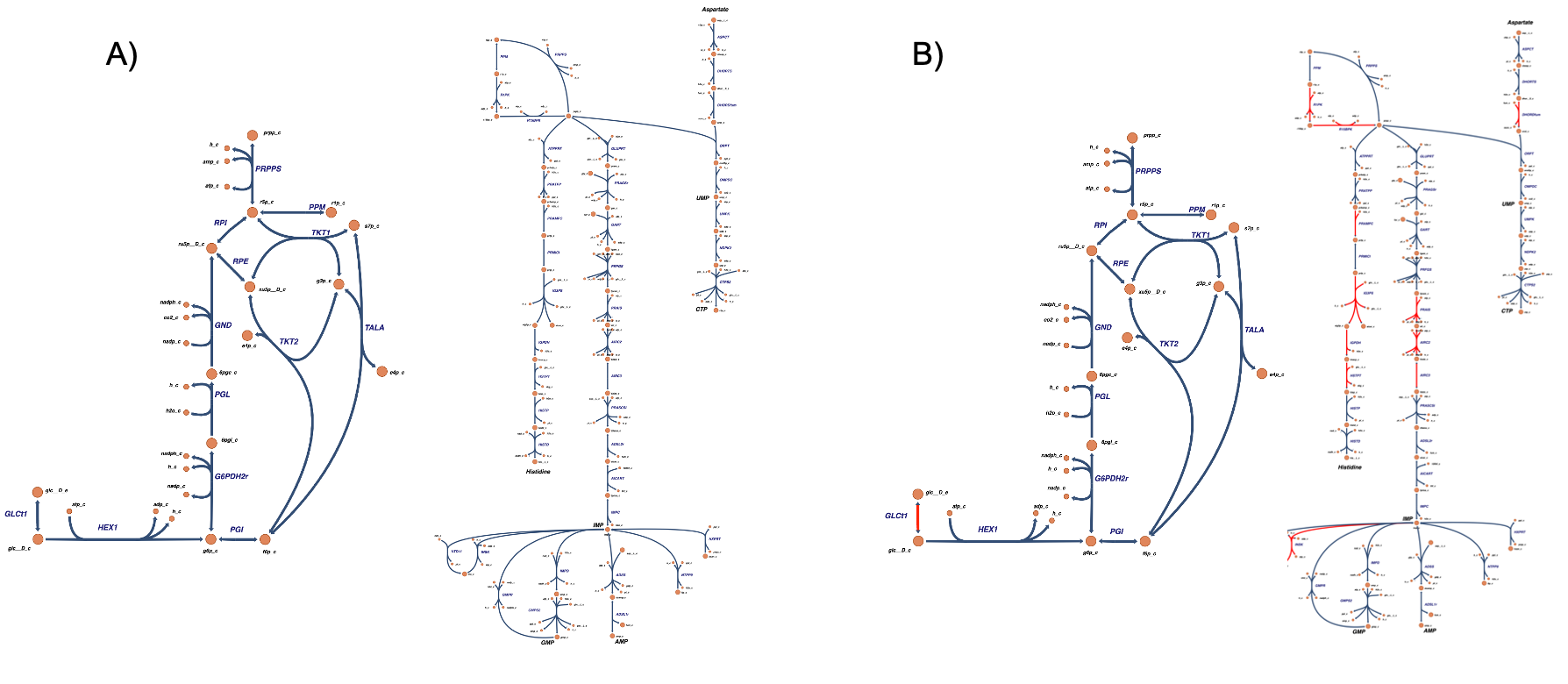


**Supplementary Figure 4:** ChiMera output visualized in Cytoscape. (A) Metabolic network of the entire organism. (B) Resulting Compounds and reactions associated with a search for one carbon by folate pathway.


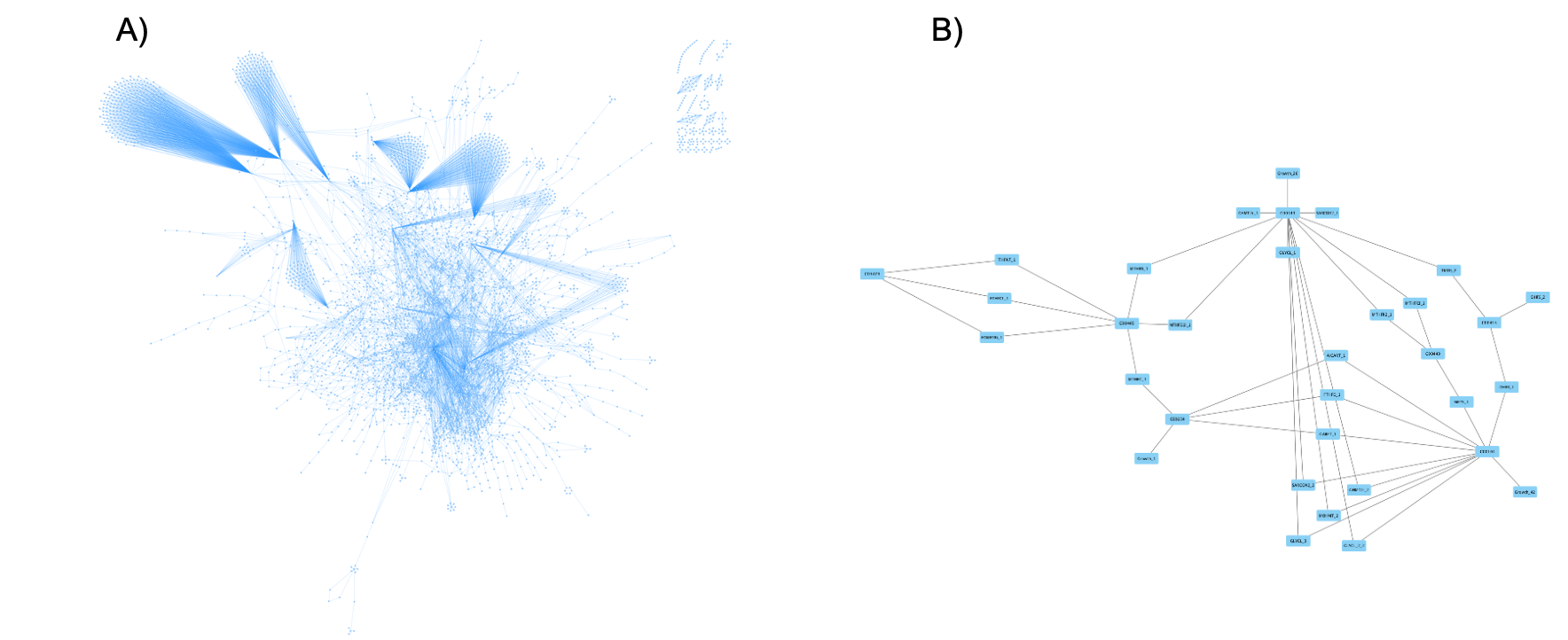


**Supplementary Figure 5:** Enriched pathways for model created by ChiMera pipeline. The 30 most abundant pathways related to the model are displayed to the user.


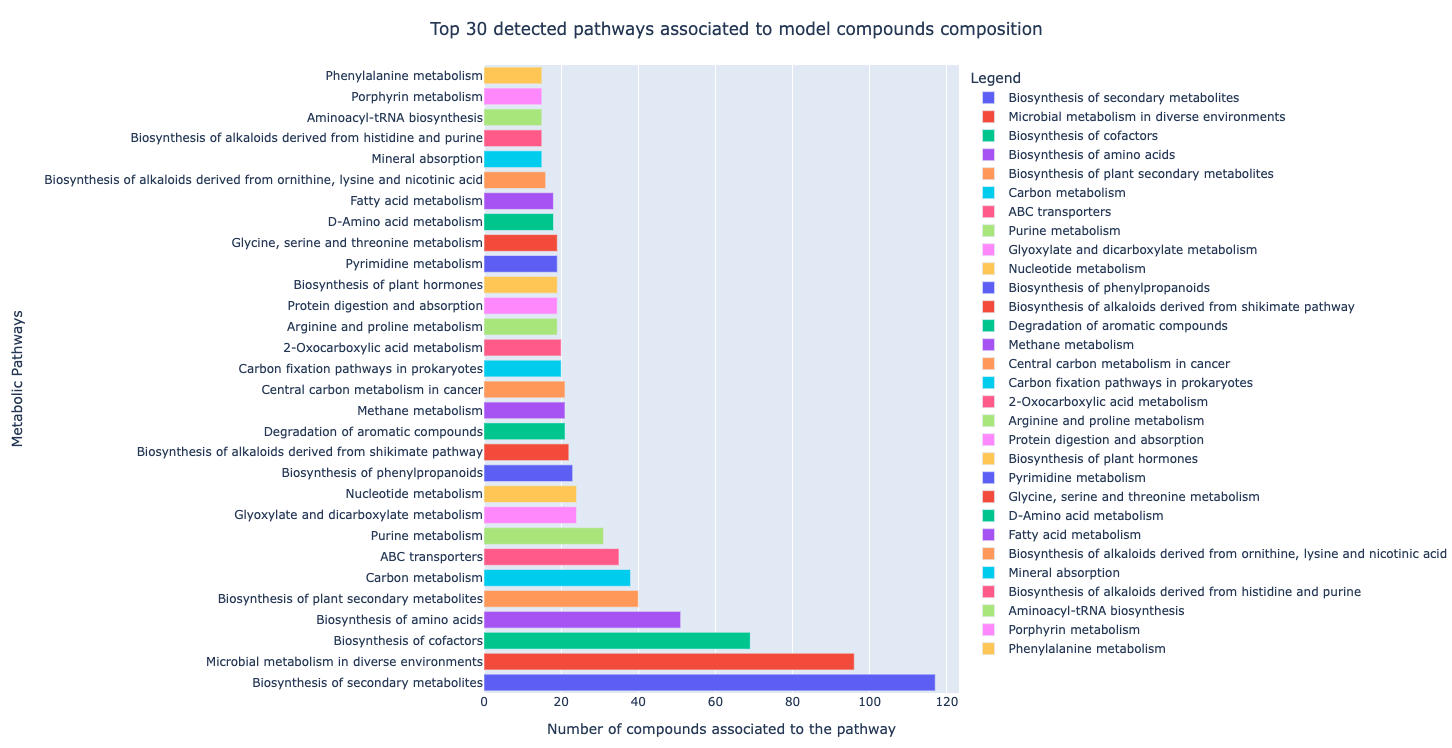

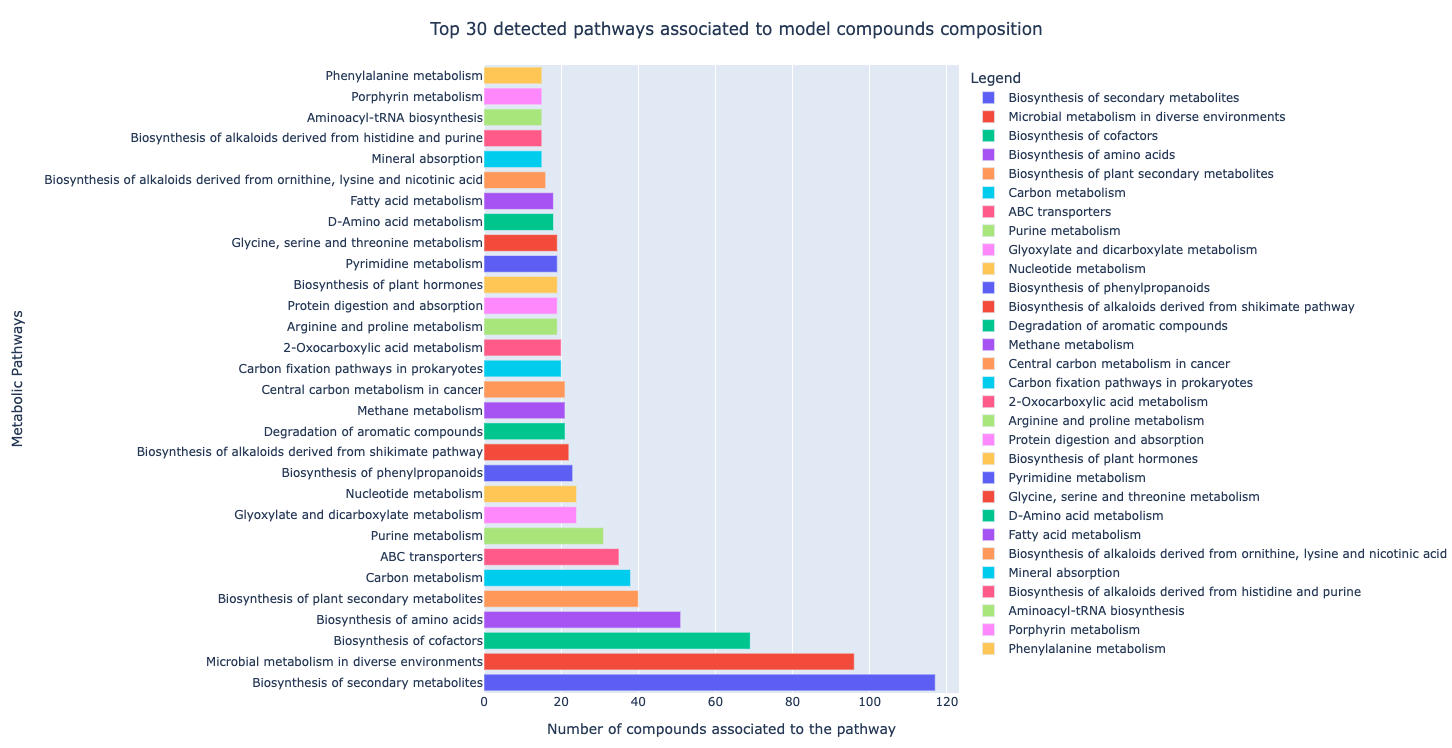

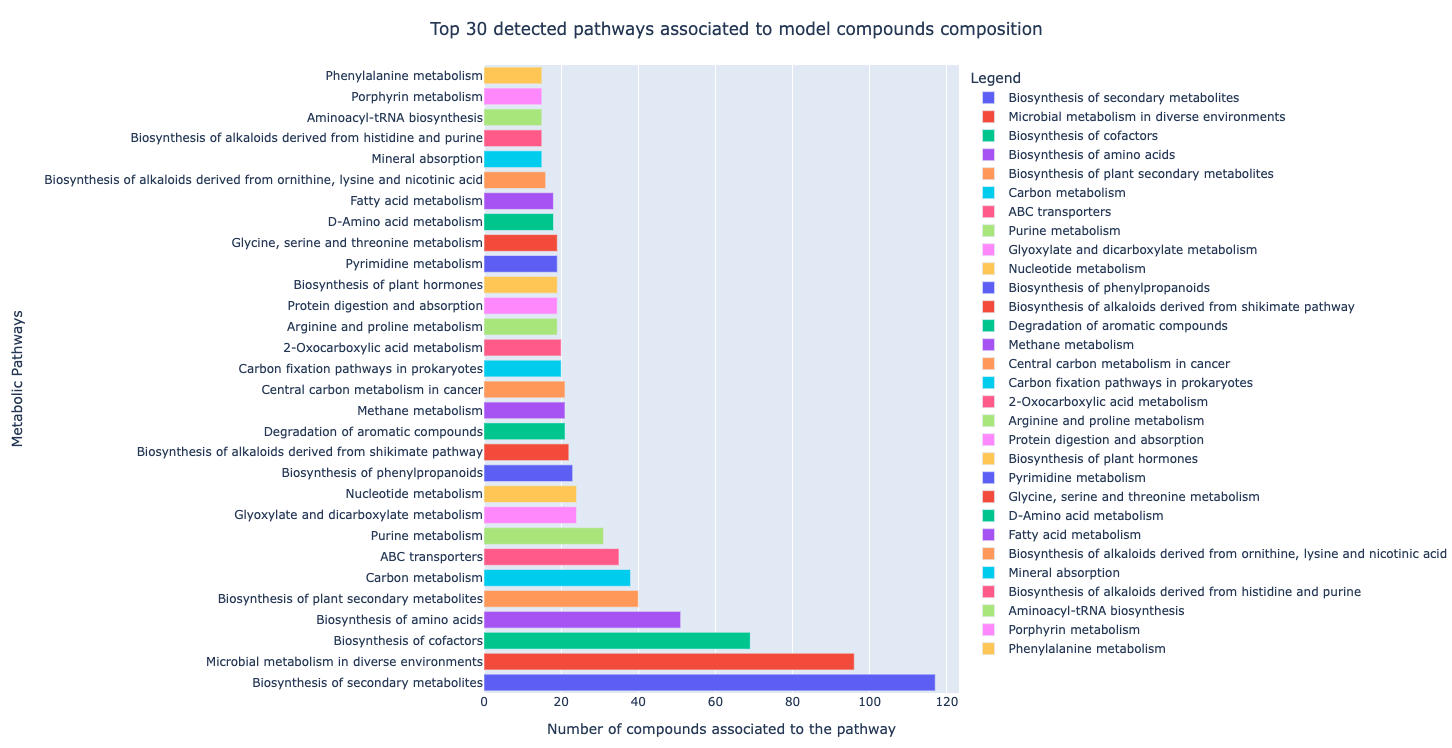


**Supplementary Figure 6:** ChiMera output of single gene knockout. The pipeline prompts to the user the growth rate before and after the knockout. It also informs if the gene was not detected in the faa file.


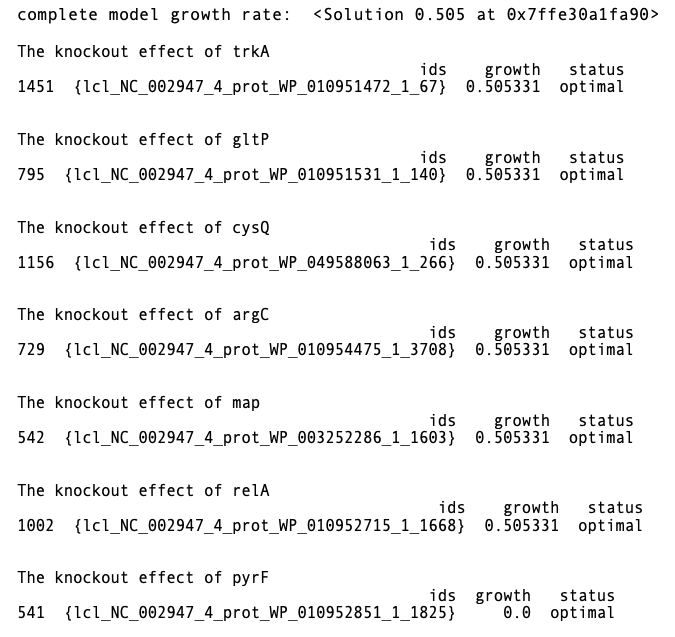


**Supplementary Figure 7:** Evaluation of model unique compounds metabolic roles. Models produced using ChiMera were compared to their relative curated model, and the set of unique compounds of each model had their metabolic association inferred using KEGG database. (A) Principal Component Analysis of models’ unique compounds. The metabolic profile of the compounds set was used in the clusterization. The legend explains the representativeness of the point coordinate in the 2D plane. (B) Clusterization of the models’ unique metabolic profile using Sq-Euclidian distance matrix. The data was log transformed and only the 30 most present pathways were used for the clusterization.
